# Clinical impact of non-alcoholic fatty liver disease on the occurrence of colorectal neoplasm: Propensity score matching analysis

**DOI:** 10.1371/journal.pone.0182014

**Published:** 2017-08-04

**Authors:** Young Joo Yang, Chang Seok Bang, Suk Pyo Shin, Gwang Ho Baik

**Affiliations:** 1 Department of Internal Medicine, Hallym University College of Medicine, Chuncheon, Korea; 2 Institue of New Frontier Research, Hallym University College of Medicine, Chuncheon, Korea; Universita degli Studi di Verona, ITALY

## Abstract

The effect of non-alcoholic fatty liver disease (NAFLD) on the occurrences of colorectal neoplasm (CRN) at surveillance colonoscopy is rarely evaluated. We retrospectively reviewed medical records of 1,023 patients who had both index and surveillance colonoscopy at a single institution. The cumulative occurrence rates of overall and advanced CRN at the time of surveillance colonoscopy were compared between patients with and without NAFLD using propensity score matching analysis. In an analysis of matched cohort of 441 patients, the cumulative rates of overall CRN occurrence at 3 and 5 years after index colonoscopy were higher in subjects with NAFLD than in those without NAFLD (9.1% *vs*. 5.0% & 35.2% *vs*. 25.3%, *P* = 0.01). Cox regression analysis showed that NAFLD independently increased the risk of overall CRN occurrence with marginal significance (adjusted hazard ratio [aHR]: 1.31 95% CI: 1.01–1.71, *P* = 0.05). Additionally, NAFLD was associated with the development of 3 or more adenomas at the time of surveillance colonoscopy (aHR: 2.49, 95% CI: 1.20–5.20, *P* = 0.02). In subgroup analysis based on index colonoscopy risk categories, the effect of NAFLD on the overall CRN occurrence at the time of surveillance colonoscopy was confined to the normal group (aHR: 1.47, 95% CI: 1.05–2.06, *P* = 0.02). Regarding advanced CRN occurrences at the time of surveillance colonoscopy, age was the only significant risk factor (aHR: 1.06, 95% CI: 1.02–1.10, *P* = 0.001). NAFLD was associated with overall CRN occurrence, especially in patients with no adenoma at the index colonoscopy. NAFLD may be considered for the determination of the time-interval for surveillance colonoscopy, especially the patients with negative index colonoscopy findings.

## Introduction

A screening colonoscopy is established as an essential method to reduce the incidence of colorectal cancer (CRC) and cancer-related mortality by removing precancerous polyps [1, 2]. A colonoscopy is highly recommended for patients who were diagnosed with or had colorectal neoplasia (CRN) resected due to high risk of metachronous or recurrent adenomatous lesions and CRC [3, 4]. Hence, current guidelines from American and European societies commonly recommend surveillance colonoscopy at determined intervals based on the findings of the most recent colonoscopy including the maximum size of the polyps, the number of adenomas, and the histologic features of CRN [5, 6].

According to previous literature, more than 75–95% of CRC developed in individuals with little or no genetic risk factors. Several dietary and lifestyle factors including obesity, high fat and low fiber diets, red meat consumption, and physical inactivity were found to be risk factors for the development of CRC [7–10]. Furthermore, metabolic syndrome, which shares common risk factors with CRC, has been investigated in terms of the effect on the development of CRN or CRC. Several studies have shown that metabolic syndrome significantly increased the risk of CRC or CRN occurrence [11, 12]. On the basis of this evidence, the modification of current post-polypectomy surveillance intervals in consideration of personalized risk factors has been suggested [12].

Non-alcoholic fatty liver disease (NAFLD) is the most common cause of chronic liver disease in Western countries, and its incidence has been increasing in Asian countries, which causes substantial economic burden [13, 14]. Some portion of NAFLD is associated with the development of not only liver cirrhosis and hepatocellular carcinoma, but also cardiovascular disease and extrahepatic malignancy [15–18]. Recently, NAFLD was suggested as the precursor of metabolic syndrome and diabetes. Because the pathogenic factors of NAFLD including insulin resistance, abdominal obesity influence the CRN development, several studies investigated the association between NAFLD and CRN [19, 20]. However, conflicting results have been reported, and the cumulative effect of NAFLD on the development of CRN at the time of surveillance colonoscopy was rarely evaluated [20–23]. Consequently, there has been controversy whether NAFLD can be considered as one of the factors to determine the optimal time interval of surveillance colonoscopy.

Although a recently published retrospective cohort study from Taiwan reported increased risk of colorectal adenoma at the 2^nd^ surveillance colonoscopy in patients with NAFLD, this study only evaluated the outcome of a specific time point and included patients with negative-risk (no adenoma) at baseline colonoscopy, omitting patients with adenoma at baseline colonoscopy [21]. To this end, we performed this study to identify the effect of NAFLD on the development of CRN at the time of surveillance colonoscopy, including all the relevant subjects regardless of the findings of index colonoscopy.

## Materials and methods

### Study population

We screened the medical records of 4,578 patients who underwent index colonoscopy between January 2009 and December 2013 at Chuncheon Sacred Heart Hospital. Among them, 1,887 patients who underwent surveillance colonoscopy after index colonoscopy were eligible for this study. Of these 1,887, 864 patients were excluded because of 1) a prior history of colorectal surgery or colorectal disease including cancer (*n* = 34), inflammatory bowel disease (*n* = 18), 2) an incomplete index colonoscopy (*n* = 48), 3) any colonoscopy within the previous 3 years of the index colonoscopy (*n* = 175), 4) chronic liver disease (*n* = 48), including chronic hepatitis B, hepatitis C or liver cirrhosis, 5) significant alcohol consumption (male >30 g/day and female >20 g/day, *n* = 159), 6) no diagnostic examination for NAFLD such as liver biopsy or abdominal image tests including computed tomography (CT), ultrasound (US), or magnetic resonance imaging within 3 months of the index colonoscopy (*n* = 370), 7) incomplete clinical information (missing variables ≥2, *n* = 12).

**Fig 1:**
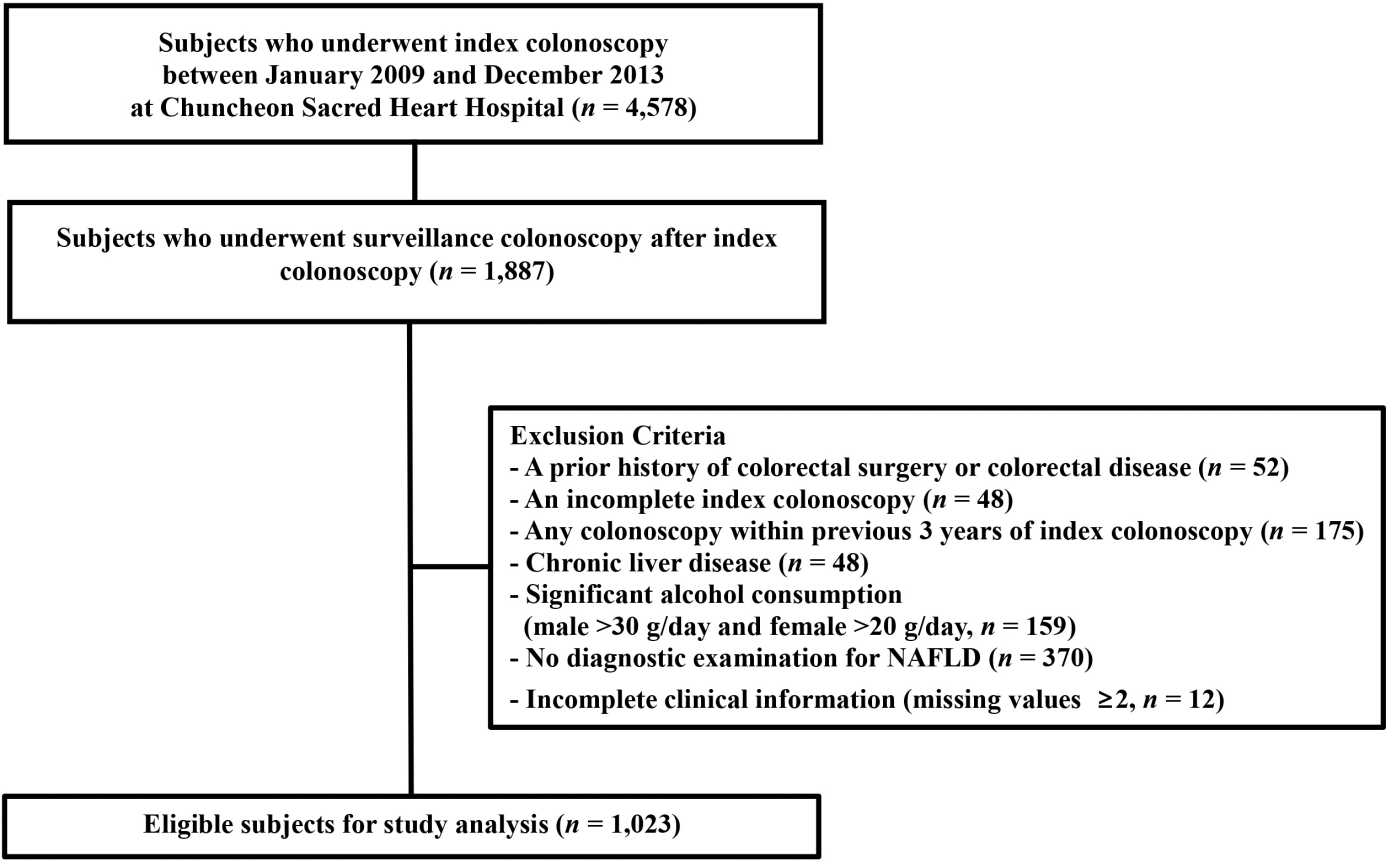
Flow chart of the study population. NAFLD, non-alcoholic fatty liver disease.

Overall, 1,023 patients were enrolled in the final analysis before propensity score matching (PSM) (Fig 1). This study was approved by the institutional review boards of Chuncheon Sacred Heart Hospital (2017–40) and all data were fully anonymized and institutional review boards waived the requirement for informed consent.

### Definitions

Surveillance colonoscopy was defined as any follow-up colonoscopic examination after index colonoscopy. To evaluate the effect of index colonoscopic findings on the development of CRN at the time of surveillance colonoscopy, index colonoscopic findings were classified into normal, low-risk, and high-risk groups. The individuals with negative index colonoscopy (no adenoma), and with 1 or 2 adenomas <10 mm were included in the normal group and the low-risk group, respectively. The individuals with advanced adenoma and/or 3 or more adenomas were considered the high-risk group. Advanced adenoma was defined as adenomas with villous or tubulo-villous histology, high grade dysplasia, or size ≥10 mm [5, 6]. Hyperplastic polyps and inflammatory polyps were regarded as normal finding. The right colon was defined as proximal to the splenic flexure, and the left colon was defined as distal to the splenic flexure including the rectum. Overall CRN included cancer or any adenomas and advanced CRN included cancer or advanced adenoma at the time of surveillance colonoscopy.

### Colonoscopy procedure

All the index and surveillance colonoscopic examinations were performed by experienced gastroenterologists after bowel preparation using 4 L of Polyethylene glycol solution. A complete colonoscopy was defined as achievement of cecal intubation and optimal withdrawal time (≥6 minutes) under adequate bowel preparation. All the CRNs found at the time of index or surveillance colonoscopy were endoscopically resected simultaneously or within 6 months after index or surveillance colonoscopy.

### Data collection and clinical outcomes

We collected the patients’ demographic characteristics and clinical information from electronic medical records, including sex, age, body mass index (BMI), smoking habits, family history of CRC in first-degree relatives, any comorbidities and medication use including aspirin or non-steroidal anti-inflammatory drugs (NSAIDs) or lipid lowering agents at the time of the index colonoscopy. Smoking habits were classified as ex- or current smoker and never smoker. The use of aspirin, NSAIDs, or lipid lowering agents was defined as taking these drugs at least twice a week for the preceding 12 months. We also checked the results of biochemical tests such as serum aspartate aminotransferase, alanine aminotransferase, fasting glucose level, and lipid profile using standard laboratory procedures at the time of the index colonoscopy. If biochemical assessment was not performed at the time of the index colonoscopy, we substituted the latest results within 3 months of the index colonoscopy.

NAFLD was defined as the cases with fatty liver and without any competing cause of fatty liver such as significant alcohol consumption, viral hepatitis, steatogenic drug. Fatty liver was detected with abdominal US or CT from experienced radiologists or gastroenterologists using standard criteria: 1) diffuse increased hepatic echogenicity: 2) exaggeration of the difference in echogenicity between liver and kidney: 3) blurring of vascular structure, and 4) lower hepatic parenchymal attenuation than that of the spleen on non-enhanced computed tomography.

Finally, we identified the number, size, location and histologic results of polyps at index and surveillance colonoscopies. Additionally, the time interval between index and surveillance colonoscopy was identified. The main clinical outcome of this study were the cumulative probabilities of overall CRN or advanced CRN on any surveillance colonoscopies. The risk factors for the development of overall CRN or advanced CRN were also analyzed.

### Statistical analysis

Continuous variables are expressed as the mean ± standard deviation. Categorical variables are expressed as number and percentage. First, we compared the differences in the baseline characteristics of the enrolled population using chi-square tests and Student’s *t*-tests for categorical and continuous variables, respectively. Then, we performed PSM to adjust potential confounding effects according to the differences in baseline characteristics between the NAFLD group and the control group. The variables used in the PSM were age, sex, BMI, current or ex- smoker, diabetes, hypertension, and the use of aspirin or NSAIDs or lipid lowering agents. The absolute standardized differences were used to diagnose the balance after matching, and all standardized mean differences after matching were less than 0.1. In the PS matched cohort, we evaluated the cumulative probabilities for the time-dependent primary outcomes using the Kaplan-Meier method and a Cox proportional hazards regression model to identify independent risk factors associated with the outcome variables. In the present study, a *P* value <0.05 (2-tailed) was adopted as the threshold for statistical significance for all tests. All of the analyses were performed using SPSS version 22.0. (SPSS Inc., Chicago, IL, USA). The detailed information for the statistical analyses of this study can be found in the S1 File.

## Results

### Baseline characteristics

The baseline characteristics at the time of index colonoscopy in unmatched and matched cohorts are presented in Table 1. We identified a total of 1,023 patients, consisting of 441 (43.1%) in the NAFLD group and 582 (56.9%) in the control group. The proportion of males (59.6% *vs*. 44.7%, *P* <0.001) was significantly higher and age (53.8 ± 10.4 *vs*. 55.1 ± 11.0, *P* = 0.05) was marginally younger in the NAFLD group than in the control group. Additionally, the patients with NAFLD had higher BMI (25.8 ± 3.6 *vs*. 23.7 ± 3.10, *P* <0.001), prevalence of current or ex-smoker (27.7% *vs*. 18.1%, *P* <0.001), hypertension (35.4% *vs*. 26.7%, *P* = 0.002), and diabetes (17.2% *vs*. 8.8%, *P* <0.001) than the control group. The proportion with a family history of CRC was not different between the two groups (2.1% *vs*. 1.4%, *P* = 0.29). The use of lipid lowering agents was significantly higher in patients with NAFLD (21.4% *vs*. 13.3%, *P* <0.001), whereas the use of aspirin or NSAIDs was comparable (18.6% *vs*. 15.1%, *P* = 0.08). At the time of index colonoscopy, 725 (70.9%), 211 (20.6%), and 87 (8.5%) patients were categorized as normal, low-risk and high-risk, respectively. The distributions of baseline risk categories and the number and location of CRN at index colonoscopy were not significantly different between the NAFLD and control groups. Also, the mean interval between the index and surveillance colonoscopy was comparable between the two groups (52.2 ± 15.1 *vs*. 51.8 ± 15.0 months, *P* = 0.70).

**Table 1:** Baseline characteristics at the time of index colonoscopy in unmatched and matched cohort.

Variables	Total population (n = 1,023)	Unmatched population			PS matched population		
		With NAFLD (n = 441)	Without NAFLD (n = 582)	P value	With NAFLD (n = 441)	Without NAFLD (n = 441)	P value
**Sex (Men)**	523 (51.1%)	263 (59.6%)	260 (44.7%)	<0.001	263 (59.6%)	228 (51.7%)	0.02
**Age (Years)**	54.6 ± 10.7	53.8 ± 10.4	55.1 ± 11.0	0.05	53.8 ± 10.4	54.5 ± 10.6	0.32
**BMI**	24.6 ± 3.5	25.8 ± 3.6	23.7 ± 3.10	<0.001	25.8 ± 3.4	24.5 ± 2.7	<0.001
**Smoking**				<0.001			0.03
**Current or ex-smoker**	226/1016 (22.2%)	122/440 (27.7%)	104/576 (18.1%)		122 (27.7%)	94 (21.3%)	
**Never smoker**	790/1016 (77.8%)	318/440 (72.3%)	472/576 (81.9%)		319 (72.3%)	347 (78.7%)	
**Family history of CRC**	17/1012 (1.7%)	9/439 (2.1%)	8/573 (1.4%)	0.29	9 (2.0%)	7 (1.6%)	0.80
**Comorbidities**							
**Hypertension**	311/1021 (30.5%)	156/441 (35.4%)	155/580 (26.7%)	0.002	156 (35.4%)	130 (29.5%)	0.07
**Diabetes**	127/1021 (12.4%)	76/441 (17.2%)	51/580 (8.8%)	<0.001	76 (17.2%)	48 (10.9%)	0.009
**Aspirin or NSAIDs use**	170/1023 (16.6%)	82/441 (18.6%)	88/582 (15.1%)	0.08	82 (18.6%)	71 (16.1%)	0.37
**Lipid lowering agent**	171/1018 (16.8%)	94/439 (21.4%)	77/579 (13.3%)	<0.001	94 (21.3%)	68 (15.4%)	0.03
**Blood indices**							
**AST (IU/L)**	26.9 ± 15.0	29.2 ± 17.8	25.2 ± 12.1	<0.001	29.2 ± 17.8	25.4 ± 12.2	<0.001
**ALT (IU/L)**	26.1 ± 20.3	30.8 ± 23.2	22.5 ± 16.9	<0.001	30.7 ± 23.2	23.5 ± 17.9	<0.001
**Fasting glucose (mg/dL)**	106.3 ± 31.0	108.9 ± 31.3	104.3 ± 30.6	0.02	108.9 ± 31.3	105. 4± 31.1	0.09
**Total cholesterol (mg/dL)**	184.4 ± 35.7	187.5 ± 36.1	182.0 ± 35.2	0.02	187.5 ± 36.1	181.6 ± 35.3	0.02
**LDL (mg/dL)**	107.5 ± 29.5	108.9 ± 30.5	106.6 ± 28.9	0.32	108.9 ± 30.5	106.6 ± 30.0	0.37
**HDL (mg/dL)**	52.0 ± 12.8	50.3 ± 11.3	53.4 ± 13.8	<0.001	50.4 ± 11.1	52.2 ± 13.1	0.05
**TG (mg/dL)**	133.9 ± 92.8	152.5 ± 106.9	118.7 ± 76.2	<0.001	152.5 ± 106.9	122.5 ± 75.1	<0.001
**Abdominal image work-up**				<0.001			<0.001
**Abdomen US**	553 (54.1%)	322 (73.0%)	231 (39.7%)		322 (73.0%)	176 (39.9%)	
**Abdomen CT**	470 (45.9%)	119 (27.0%)	351 (60.3%)		119 (27.0%)	265 (60.1%)	
**Index colonoscopy findings**				0.65			0.43
**normal**	725 (70.9%)	318 (72.1%)	407 (69.9%)		318/441 (72.1%)	307/441 (69.6%)	
**low risk**	211 (20.6%)	85 (19.3%)	126 (21.6%)		85/441 (19.3%)	100/441 (22.7%)	
**high risk**	87 (8.5%)	38 (8.6%)	49 (8.4%)		38/441 (8.6%)	34/441 (7.7%)	
**Number of CRN**				0.97			0.85
**1 adenoma**	182 (61.1%)	76 (61.8%)	106 (60.6%)		76/123 (61.8%)	80/134 (59.7%)	
**2 adenomas**	68 (22.8%)	27 (22.0%)	41 (23.4%)		27/123 (22.0%)	32/134 (23.9%)	
**3 or more adenomas**	48 (16.1%)	20 (16.3%)	28 (16.0%)		20/123 (16.3%)	22/134 (16.4%)	
**location of CRN**				0.74			0.66
**Left colon**	109 (36.6%)	48 (39.0%)	61 (34.9%)		48/123 (39.0%)	45/134 (33.6%)	
**Right colon**	120 (40.3%)	47 (38.2%)	73 (41.7%)		47/123 (38.2%)	57/134 (42.5%)	
**Both colon**	69 (23.2%)	28 (22.8%)	41 (23.4%)		28/123 (22.8%)	32/134 (23.9%)	
**Interval from baseline to last surveillance colonoscopy (months)**	52.0 ± 15.1	52.2 ± 15.1	51.8 ± 15.0	0.70	52.2 ± 15.1	51.8 ± 15.2	0.71

NAFLD, nonalcoholic fatty liver disease; BMI, body mass index; CRC, colorectal cancer; NSAID, non-steroidal anti-inflammatory drugs; AST, aspartate aminotransferase; ALT, alanine aminotransferase; LDL, low density lipid; HDL, high density lipid; TG, triglyceride; CRN, colorectal neoplasm

PSM analysis identified 441 patients in the NAFLD group and 441 patients in the control group, and all the variables used in PSM were well-balanced (standardized mean differences after matching were less than 0.1 in all the confounding variables used in the matching: age, sex, BMI, current or ex- smoker, diabetes, hypertension, and the use of aspirin or NSAIDs or lipid lowering agents). After PSM, the differences in mean age, the prevalence of hypertension and the mean level of fasting glucose between the NAFLD and control groups were no longer statistically significant. In PS matched cohorts, abdomen US and CT were performed in 322 (73.0%) and 119 (27.0%) of 441 patients with NALFD whereas in 441 patients without NAFLD, 176 (39.9%) and 265 (60.1%) patients performed abdomen US and CT, respectively.

### Cumulative incidence and risk factors for overall CRN occurrence in the matched cohort

**Fig 2:**
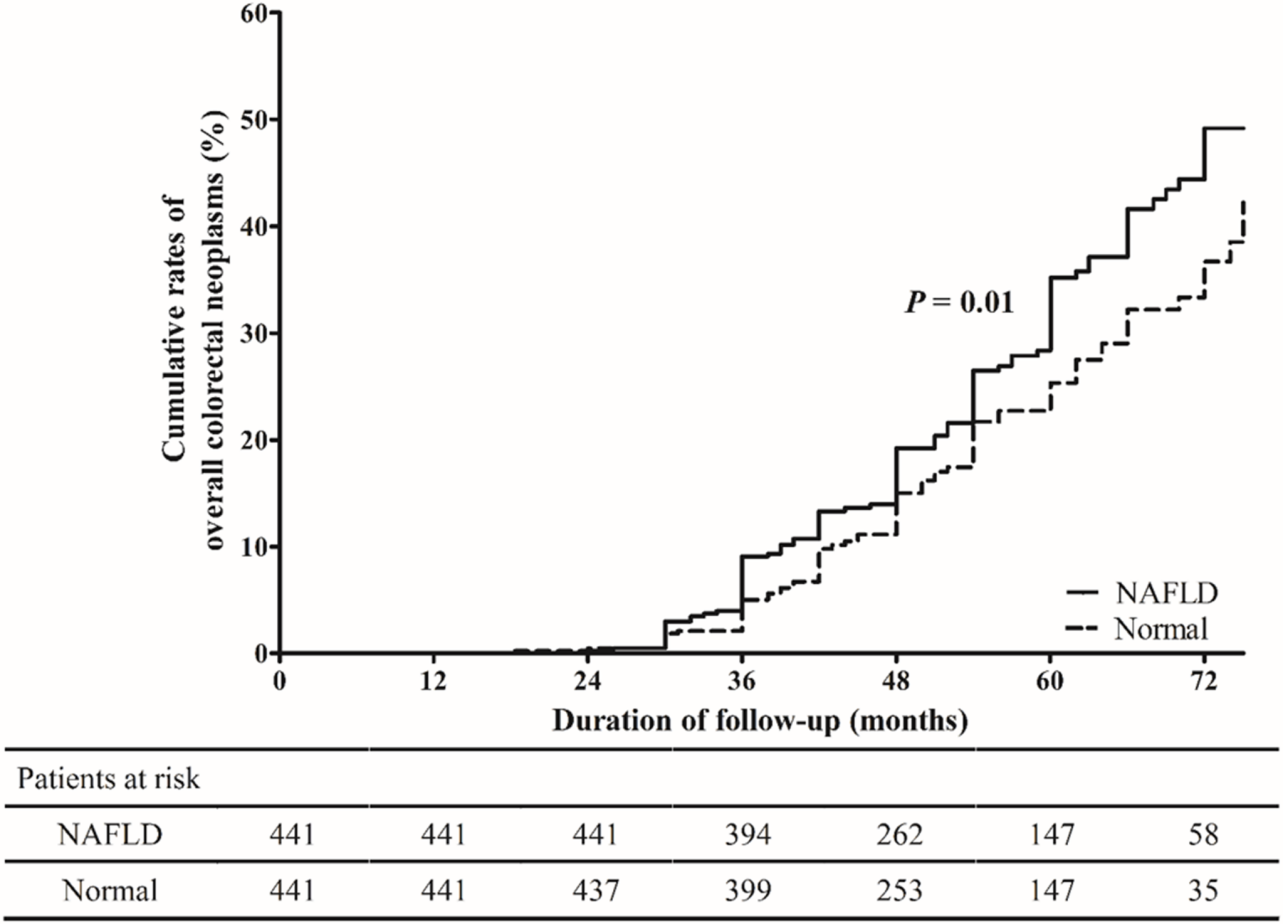
Cumulative rates of overall CRN occurrence in the NAFLD and normal groups at the time of the surveillance colonoscopy. CRN, colorectal neoplasm; NAFLD, non-alcoholic fatty liver disease.

Among the 882 matched cohort patients, overall CRN developed in 135 (30.6%) patients with NAFLD and 99 (22.4%) patients without NAFLD at the time of surveillance colonoscopy (*P* = 0.008). The cumulative rates of overall CRN at 3 and 5 years in the NAFLD group were 9.1% and 35.2%, respectively, which were significantly higher than those of the control group (5.0% and 25.3%, *P* = 0.01) (Fig 2). Univariate and subsequent multivariate Cox regression analysis showed that NAFLD was an independent risk factor for the occurrence of overall CRN with marginal significance (adjusted hazard ratio [aHR]: 1.31, 95% confidence interval [CI]: 1.01–1.71, *P* = 0.05), along with male gender (aHR: 1.63, 95% CI: 1.23–2.15, *P* = 0.001), old age (aHR: 1.02, 95% CI: 1.01–1.04, *P* = 0.001), and the presence of diabetes (aHR: 1.59, 95% CI: 1.15–2.21, *P* = 0.006) (Table 2).

**Table 2:** Univariate and multivariate analyses for the risk factors of overall CRN at the surveillance colonoscopy in matched cases using propensity scores.

Variables	Univariate analysis		Multivariate analysis*	
	HR (95% CI)	*P* value	HR (95% CI)	*P* value
**Sex (Men)**	1.68 (1.28–2.21)	< 0.001	1.63 (1.23–2.15)	0.001
**Age**	1.03 (1.01–1.04)	< 0.001	1.02 (1.01–1.04)	0.001
**BMI (kg/m**^**2**^**)**	1.02 (0.98–1.06)	0.35		
**Current or ex- Smoking**	1.39 (1.06–1.84)	0.02	1.10 (0.80–1.52)	0.55
**Family history of CRC**	1.10 (0.41–2.97)	0.85		
**Hypertension**	1.46 (1.12–1.90)	0.005	1.12 (0.84–1.50)	0.45
**Diabetes**	2.02 (1.47–2.77)	< 0.001	1.59 (1.15–2.21)	0.006
**Aspirin or NSAIDs use**	1.27 (0.91–1.77)	0.16	0.85 (0.56–1.28)	0.44
**Lipid lowering agent**	1.26 (0.92–1.73)	0.15	0.84 (0.58–1.20)	0.34
**Abdomen US (reference) vs. CT**	0.84 (0.64–1.11)	0.22	0.90 (0.67–1.20)	0.47
**NAFLD**	1.40 (1.08–1.81)	0.01	1.31 (1.01–1.71)	0.05
**Index colonoscopy findings**				
**Normal risk**	reference		reference	
**Low risk**	1.60 (1.18–2.16)	0.002	1.35 (0.99–1.84)	0.06
**High risk**	1.74 (1.16–2.60)	0.007	1.42 (0.94–2.14)	0.09
**Adenoma ≥3 at index colonoscopy**	1.62 (0.99–2.66)	0.06	0.96 (0.44–2.09)	0.92

BMI, body mass index; CRC, colorectal cancer; NSAID, non-steroidal anti-inflammatory drugs; NAFLD, nonalcoholic fatty liver disease; US, ultrasound; CT, computed tomography; HR: hazard ratio, CI: confidence interval.

All variables with *P* ≤0.3 by univariate analysis were analyzed by multivariate analysis.

*Adjusted for sex, age, current or ex-smoking, hypertension, Diabetes, aspirin or NSAIDs use, lipid lowering agent, Abdomen US (reference) vs. CT, NAFLD, risk categories based on index colonoscopy findings, and Adenoma ≥ 3 at index colonoscopy.

### Association between NAFLD and multiplicity of CRN at the surveillance colonoscopy

**Fig 3:**
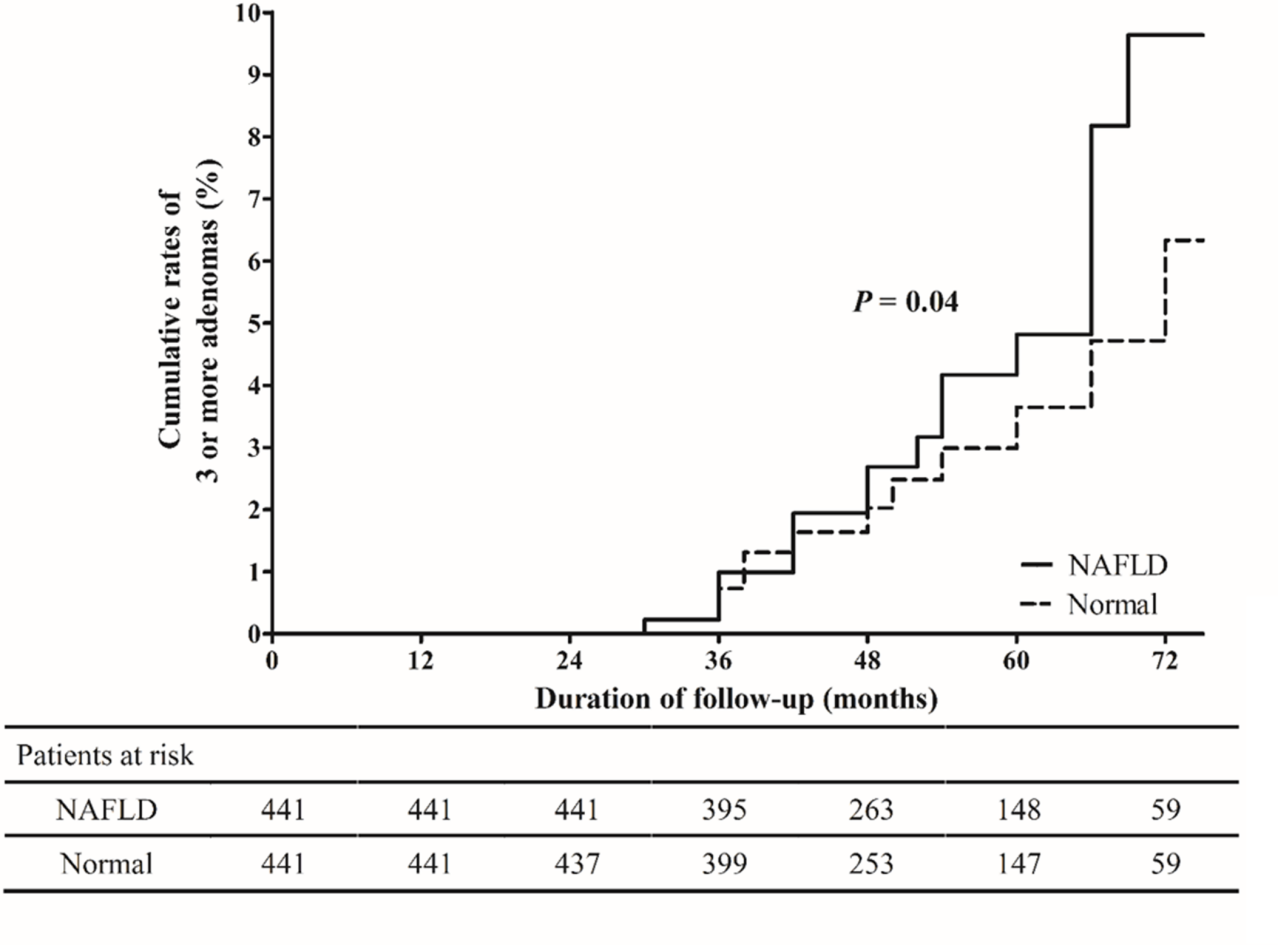
Cumulative rates of 3 or more adenomas in the NAFLD and normal groups at the time of the surveillance colonoscopy. NAFLD, non-alcoholic fatty liver disease.

The occurrence of 3 or more adenomas at the surveillance colonoscopy was observed in 23 (5.2%) patients with NAFLD and 12 (2.7%) patients without NAFLD (*P* = 0.08). The cumulative rates of 3 or more adenomas at 3 and 5 years after index colonoscopy were significantly higher in subjects with NAFLD than in the subjects without NAFLD (1.0% *vs*. 0.7% & 4.8% *vs*. 3.6%, *P* = 0.04) (Fig 3). In a multivariate analysis, NAFLD (aHR: 2.49, 95% CI: 1.20–5.20, *P* = 0.02) increased the risk of the development of 3 or more adenomas at the surveillance colonoscopy along with male gender (aHR: 2.75, 95% CI: 1.27–5.96, *P* = 0.01), old age (aHR: 1.05, 95% CI: 1.02–1.09, *P* = 0.002), and the performance of abdomen CT for the diagnosis of NAFLD (aHR: 2.08, 95% CI: 1.03–4.22, *P* = 0.04) (Table 3).

**Table 3:** Univariate and multivariate analysis of risk factors for adenoma ≥3 at the surveillance colonoscopy in matched cases using propensity scores.

	Univariate analysis		Multivariate analysis*	
**Variables**	**HR (95% CI)**	***P* value**	**HR (95% CI)**	***P* value**
**Sex (male)**	2.47 (1.15–5.28)	0.02	2.75 (1.27–5.96)	0.01
**Age**	1.05 (1.01–1.08)	0.007	1.05 (1.02–1.09)	0.002
**BMI (kg/m**^**2**^**)**	1.04 (0.94–1.15)	0.45		
**Current or ex- Smoking**	2.09 (1.06–4.13)	0.03	1.32 (0.60–2.94)	0.49
**Family history of CRC**	2.20 (0.30–16.21)	0.44		
**Hypertension**	1.65 (0.84–3.24)	0.14	1.06 (0.50–2.23)	0.88
**Diabetes**	2.75 (1.27–5.93)	0.01	1.95 (0.88–4.35)	0.10
**Aspirin or NSAIDs use**	1.48 (0.64–3.41)	0.36		
**Lipid lowering agent**	1.09 (0.48–2.54)	0.83		
**Abdomen US (reference) vs. CT**	1.59 (0.81–3.12)	0.18	2.08 (1.03–4.22)	0.04
**NAFLD**	2.01 (1.00–4.05)	0.05	2.49 (1.20–5.20)	0.02
**Index colonoscopy findings**				
**Normal risk**	reference		reference	
**Low risk**	2.75 (1.29–5.84)	0.09	2.20 (1.00–4.94)	0.05
**High risk**	3.28 (1.32–8.20)	0.01	1.93 (0.72–5.17)	0.19
**Adenoma ≥3 at index colonoscopy**	3.40 (1.30–8.89)	0.01	5.42 (0.66–44.35)	0.12

BMI, body mass index; CRC, colorectal cancer; NSAID, non-steroidal anti-inflammatory drugs; NAFLD, nonalcoholic fatty liver disease; US, ultrasound; CT, computed tomography; HR: hazard ratio, CI: confidence interval.

All variables with *P* ≤0.3 by univariate analysis were analyzed by multivariate analysis.

* Adjusted for sex, age, current or ex-smoking, hypertension, Diabetes, Abdomen US (reference) vs. CT, NAFLD, risk categories based on index colonoscopy findings, and Adenoma ≥ 3 at index colonoscopy

### Subgroup analysis of overall CRN occurrence according to risk stratification based on the findings at the index colonoscopy

We evaluated the independent risk factors of overall CRN occurrence according to the risk stratification based on the findings at the index colonoscopy. In the normal group, NAFLD was significantly associated with overall CRN occurrence at the time of the surveillance colonoscopy (aHR: 1.47, 95% CI: 1.05–2.06, *P* = 0.02), and male gender and old age were also significant risk factors (aHR: 1.85, 95% CI: 1.31–2.63, *P* = 0.001, and aHR: 1.03, 95% CI: 1.01–1.05, *P* <0.001). In the low-risk group, BMI (aHR: 1.12, 95% CI: 1.04–1.20, *P* = 0.003) and diabetes (aHR: 1.96, 95% CI: 1.08–3.57, *P* = 0.03) were associated with overall CRN occurrence. In the high-risk group, diabetes (aHR: 3.46, 95% CI: 1.51–7.91, *P* = 0.003) and low BMI (aHR: 0.88, 95% CI: 0.80–0.98, *P* = 0.02) were significant risk factors for overall CRN occurrence (Table 4).

**Table 4:** Multivariate analysis for the risk factors of overall CRN according to risk stratification based on the findings at index colonoscopy.

	Normal				Low risk				High risk			
	Univariate analysis		Multivariate analysis*		Univariate analysis		Multivariate analysis^†^		Univariate analysis		Multivariate analysis^‡^	
Variables	HR (95% CI)	*P* value	HR (95% CI)	*P* value	HR (95% CI)	*P* value	HR (95% CI)	*P* value	HR (95% CI)	*P* value	HR (95% CI)	*P* value
**Sex (male)**	1.76 (1.25–2.49)	0.001	1.85 (1.31–2.63)	0.001	1.14 (0.67–1.94)	0.63			2.25 (0.83–6.08)	0.11	1.66 (0.60–4.59)	0.33
**Age**	1.03 (1.01–1.04)	0.003	1.03 (1.01–1.05)	<0.001	1.02 (0.99–1.04	0.29	1.00(0.97–1.03)	0.84	1.03 (0.99–1.06)	0.14	1.01 (0.97–4.68)	0.79
**BMI (kg/m**^**2**^**)**	1.00 (0.95–1.06)	0.89			1.12 (1.05–1.21)	0.001	1.12 (1.04–1.20)	0.003	0.87 (0.77–0.98)	0.02	0.88 (0.80–0.98)	0.02
**Current or ex- Smoking**	1.62 (1.13–2.32)	0.009	1.31 (0.87–1.98)	0.20	0.96 (0.55–1.68)	0.88			1.18 (0.55–2.51)	0.68		
**Family history of CRC**	1.23 (0.30–4.97)	0.77			0.05 (0–865.09)	0.54			1.06 (0.25–4.46)	0.94		
**Hypertension**	1.32 (0.94–1.85)	0.11	1.02(0.70–1.50)	0.91	1.61 (0.97–2.69))	0.07	1.25(0.65–2.39)	0.51	1.68 (0.79–3.59)	0.18	1.97 (0.84–4.50)	0.11
**Diabetes**	1.58 (1.02–2.45)	0.04	1.10(0.69–1.75)	0.69	2.22 (1.22–4.01)	0.009	1.96 (1.08–3.57)	0.03	3.11 (1.39–6.98)	0.006	3.46 (1.51–7.91)	0.003
**Aspirin or NSAIDs use**	1.03 (0.65–1.62	0.90			1.54 (0.83–2.86))	0.17	0.76(0.35–1.66)	0.49	2.30 (1.00–5.29)	0.05	1.34 (0.52–3.44)	0.55
**Lipid lowering agent**	1.06 (0.69–1.62)	0.79			2.51 (1.43–4.41)	0.001	1.54(0.77–3.09)	0.22	0.73 (0.28–1.92)	0.52		
**Abdomen US (reference) vs. CT**	0.74 (0.52–1.05)	0.10	0.84(0.58–1.20)	0.35	0.94 (0.56–1.60)	0.83			1.15 (0.55–2.44)	0.71		
**NAFLD**	1.51 (1.08–2.12)	0.02	1.47 (1.05–2.06)	0.02	1.90 (1.12–3.20)	0.02	1.56 (0.91–2.66)	0.11	0.52 (0.24–1.12)	0.09	0.94 (0.39–2.26)	0.89

BMI, body mass index; CRC, colorectal cancer; NSAID, non-steroidal anti-inflammatory drugs; NAFLD, nonalcoholic fatty liver disease; US, ultrasound; CT, computed tomography; HR: hazard ratio, CI: confidence interval.

All variables with *P* ≤0.3 by univariate analysis were analyzed by multivariate analysis.

* Adjusted for sex, age, current or ex-smoking, hypertension, Diabetes, Abdomen US (reference) vs. CT, NAFLD.

^†^ Adjusted for age, BMI, hypertension, Diabetes, Aspirin or NSAIDs use, Lipid lowering agent, NAFLD.

^‡^ Adjusted for sex, age, BMI, hypertension, Diabetes, Aspirin or NSAIDs use, NAFLD.

### Cumulative incidence and risk factors for advanced CRN occurrence in the matched cohort

A total of 30 advanced CRNs including invasive cancer (*n* = 1), high grade dysplasia (*n* = 3), adenoma ≥1cm (*n* = 25), and villous adenoma (*n* = 3) had developed at the time of the surveillance colonoscopy. Sixteen (3.6%) patients in the NAFLD group and 14 (3.2%) patients in the control group were diagnosed with advanced CRN at the surveillance colonoscopy (*P* = 0.85). The cumulative rates of advanced CRN occurrence at 3 (0.7% *vs*. 1.2%) and 5 years (4.2% *vs*. 5.4%) after index colonoscopy were comparable between the NAFLD and control groups (*P* = 0.74). Cox regression analysis identified old age (aHR: 1.06, 95% CI: 1.02–1.10, *P* = 0.001) as the only variable significantly associated with the occurrence of advanced CRN at the time of the surveillance colonoscopy (Table 5).

**Table 5:** Univariate and multivariate analysis for the risk factors of advanced CRN at surveillance colonoscopy in matched cases using propensity scores.

Variables	Univariate analysis		Multivariate analysis*	
	HR (95% CI)	*P* value	HR (95% CI)	*P* value
**Sex (male)**	1.01 (0.49–2.07)	0.99		
**Age**	1.06 (1.02–1.10)	0.001	1.06 (1.02–1.10)	0.001
**BMI (kg/m**^**2**^**)**	1.06 (0.95–1.17)	0.30	1.05 (0.95–1.17)	0.35
**Current or ex- Smoking**	0.75 (0.31–1.83)	0.53		
**Family history of CRC**	0.05 (0–16174.0)	0.64		
**Hypertension**	1.97 (0.96–4.04)	0.07	1.27 (0.57–2.84)	0.55
**Diabetes**	1.76 (0.72–4.30)	0.22	1.25 (0.49–3.18)	0.64
**Aspirin or NSAIDs use**	1.59 (0.68–3.70)	0.29	0.88 (0.33–2.39	0.81
**Lipid lowering agent**	1.67 (0.74–3.77)	0.22	1.05 (0.41–2.72)	0.92
**Abdomen US (reference) vs. CT**	0.87 (0.41–1.83)	0.71		
**NAFLD**	1.13 (0.55–2.32)	0.74	1.07(0.51–2.26)	0.85
**Index colonoscopy findings**				
**Normal risk**	Reference		Reference	
**Low risk**	2.07 (0.92–4.69)	0.08	1.56 (0.67–3.60)	1.56 (0.67–3.60)
**High risk**	2.86 (1.05–7.82)	0.04	2.34 (0.84–6.47)	0.10
**Adenoma ≥3 at index colonoscopy**	1.53 (0.36–6.44)	0.56		

BMI, body mass index; CRC, colorectal cancer; NSAID, non-steroidal anti-inflammatory drugs; NAFLD, nonalcoholic fatty liver disease; US, ultrasound; CT, computed tomography; HR: hazard ratio, CI: confidence interval.

All variables with *P* ≤0.3 by univariate analysis were analyzed by multivariate analysis.

* Adjusted for age, BMI, hypertension, Diabetes, aspirin or NSAIDs use, lipid lowering agent and risk categories based on index colonoscopy findings.

## Discussion

This study evaluated the cumulative effect of NAFLD on CRN occurrence and considered index colonoscopy risk stratification using PSM, and identified that NAFLD independently increased the risk of overall CRN occurrence at the time of the surveillance colonoscopy, especially in subjects with normal colonoscopy findings (no adenoma) at the index colonoscopy. In addition, NAFLD was associated with an increased number of adenomas at the surveillance colonoscopy. However, NAFLD had no influence on the occurrence of advanced CRN at the surveillance colonoscopy.

Previous retrospective cross-sectional studies have investigated the effect of NAFLD on the prevalence CRN. Hwang et al. reported that NAFLD was significantly associated with CRN [24], whereas Tozuin et al. reported no association [20]. In 2 recent published meta-analyses, NAFLD showed a significant effect on the occurrence of CRN, and the development of multiple CRN. However, NAFLD was not associated with CRN located in the proximal colon or, the development of advanced CRN [25, 26], which is consistent with our findings. The reason why NAFLD had no effect on the development of advanced CRN at the surveillance colonoscopy cannot be elucidated by our study. The possible explanation is that NAFLD as a single risk factor might not be sufficient to promote advanced CRN. One Taiwanese study demonstrated that metabolic syndrome significantly increased the risk of advanced CRN after negative- or low-risk findings at the baseline colonoscopy and showed the individual components of metabolic syndrome were not related to advanced CRN [12]. Similar results were also observed in a Korean study that evaluated the effect of individual metabolic factors on CRN occurrence at the surveillance colonoscopy [27]. To evaluate the association between NAFLD and advanced CRN at the surveillance colonoscopy, further well-organized prospective studies adjusting for multiple components of metabolic syndrome are needed.

Our study differs from previous studies in that we conducted PSM to find the genuine effect of NAFLD on the occurrence of CRN at the surveillance colonoscopy and included the patients with low-risk and high-risk patients based on the index colonoscopy. Huang et al. showed the effect of NAFLD on adenoma development at 2^nd^ colonoscopy only in patients with negative index colonoscopy [21]. This result was similar to our study findings. However, we found that NAFLD had little influence on the occurrence of CRN at the surveillance colonoscopy in patients with any adenoma at the index colonoscopy, which proposes the hypothesis that NAFLD could be more associated with the initiation of CRN development. Therefore, NAFLD may be considered an important risk factor in patients with negative index colonoscopy demanding surveillance colonoscopy.

Colon cancer screening programs with colonoscopies lead to economic burden, and there is also concern about procedure-related adverse events [28], emphasizing the necessity of optimal strategy or risk stratification for the subjects who require surveillance colonoscopy. Previous retrospective studies demonstrated that metabolic syndrome or its individual components independently increased the risk of CRN occurrence at surveillance colonoscopy [12, 27]. Putting these findings together with our results, surveillance colonoscopy based on tailored risk stratification is warranted considering not only traditional risk stratification according to baseline colonoscopic findings including the multiplicity, size, and histologic features, but also personalized risk factors according to well-known risk factors for CRC such as metabolic syndrome or the presence of NAFLD.

This study had a few limitations. Although the differences in the baseline characteristics of the enrolled population were adjusted by PSM in order to overcome potential confounding effects, the retrospective study design inevitably produced selection bias, and undetected variables, such as waist circumstance could influence the results. Also, we could not check the changes in NAFLD over the time, which could have some degree of influence on CRN occurrence. Second, NAFLD was mostly diagnosed by an abdominal image test such as US or CT without liver biopsy in our study. Third, the impact of sessile serrated adenoma/polyps could not be evaluated because they were not accurately identified during the study period.

In conclusion, NAFLD increased the risk of overall CRN, especially in subjects with no adenoma at index colonoscopy, and the number of adenoma at the surveillance colonoscopy. NAFLD should be included for the determination of optimal time -intervals for surveillance colonoscopy.

## Supporting information

S1 File This file contains detailed information for the statistical analyses of this study. (SAV)

## Abbreviations

NAFLD: non-alcoholic fatty liver disease
CRN: colorectal neoplasm
PSM: propensity score matching
HR: hazard ratio
CI: confidence interval
CRC: colorectal cancer
BMI: body mass index
NSAID: non-steroidal anti-inflammatory drugs
